# Identification of Swedish caries active individuals aged 30–90 years using a life course perspective and SKaPa longitudinal national registry data over a 10-year period

**DOI:** 10.2340/aos.v83.40955

**Published:** 2024-06-26

**Authors:** Håkan Flink, Anders Hedenbjörk-Lager, Simon Liljeström, Eva Nohlert, Åke Tegelberg

**Affiliations:** a Centre for Clinical Research Västerås, Uppsala University, Västerås, Sweden; b Faculty of Odontology, Malmö University, Malmö, Sweden

**Keywords:** Caries experience, caries prevention, disease progression, epidemiology, life course perspective

## Abstract

**Objective:**

To describe the occurrence of caries disease from a life course perspective using longitudinal data from the Swedish Quality Registry for Caries and Periodontal Disease (SKaPa).

**Material and Methods:**

Data from seven age cohorts (ages 30–90 years), each followed over 10 years, were retrieved from the SKaPa. Using a three-trajectory model, individuals were divided into three trajectories according to their caries development over time: high (15%), moderate (45%), or low (40%). Caries experience was expressed as the mean decayed, missing, and filled surfaces (DMFS) index.

**Results:**

Significant differences were found for all three trajectories and in all age groups over the 10 years. The mean DMFS index increase was significantly larger for the high trajectory group than for the moderate and low trajectory groups across all age cohorts. An increase in caries experience was observed for the older cohorts across all trajectories.

**Conclusions:**

A three-trajectory model appears useful for identifying and quantifying caries experiences in longitudinal studies. Increased caries disease occurs over time, especially in the highest trajectory group and among older cohorts. These findings emphasise the need for greater attention and more efficient caries prevention methods.

## Introduction

The life course perspective is a way to approach the study of chronic disease causes and risks. Throughout the life course and especially during growth, critical periods are essential to the development of tissues and organs and may affect health later in life [1, 2]. Few published studies have focussed on the relations between caries disease and developmental life course factors among adults [3–5], and thus further longitudinal studies are needed to better explain these relationships. This is especially true for individuals with untreated dental caries, the most prevalent non-communicable disease worldwide, which may affect the permanent dentition [6, 7].

The most common method for describing caries experience is the decayed, missing, and filled surfaces (DMFS) index [8]. The prevalence of caries has decreased in many countries and increasing numbers of adults now have few or no caries lesions. Although this pattern is reflected in a decreased DMFS index [9–11], the portion of the population with recurring caries lesions is obscured by the skewed distribution [12, 13].

The longest birth cohort study of caries experience in adulthood was born in Dunedin, New Zeeland, in 1972 [14, 15]. The study population was assigned to one of three caries development trajectories reflecting population differences in caries occurrence [16, 17]. Using group-based trajectory analysis to investigate the natural history of dental caries experience from childhood to middle-age showed that 15% of the population had the highest caries experience, 45% had low or limited caries, and 40% had almost no caries.

The Swedish Quality Registry for Caries and Periodontal Disease (SKaPa) includes data from all adult age groups, offering a unique way to investigate the prevalence of recurring caries disease among adults in Sweden. This is valuable because many countries lack longitudinal caries data for adults.

The SKaPa became operational in 2008 and uses automatic data retrieval directly from electronic patient dental records [18]. The database contains dental care information (including caries and periodontitis) for 7.4 million individuals including longitudinal data spanning over 10 years [10] for much of Sweden’s total population (~ 10 million in 2019) [19].

The three-trajectory caries model described by Broadbent et al. in the Dunedin study [16, 17] was previously used to analyse two cohorts of SKaPa data (the 30- and 40-year-old groups) [20], with consistent findings regarding identifying individuals with the highest caries experience over time.

The remaining question is whether a similar pattern can be discerned in other age cohorts using the three-trajectory caries model. If a general pattern is confirmed in all age cohorts, the model could be used to correlate caries trajectories with other Swedish chronic disease registers (e.g. diabetes, asthma, rheumatic diseases), potentially improving our understanding of comorbidities. A similar approach may also be useful for identifying associations with hyposalivation-causing medication use, which may influence caries disease.

By including age cohorts, the SKaPa offers a unique opportunity to investigate the caries experience within the Swedish population. These longitudinal caries data also allow us to make distinctions between those who are caries-active or caries-inactive, and to compare these data in ways not possible with cross-sectional studies.

Few studies have focussed on ongoing caries progression and its effects on daily living in adult samples [21–23]. The few available studies shows that the common wish and goal among that group is halting caries progression [21, 22]. The trajectory model may provide a way to compare differences in experiences, efforts, wishes, and goals between caries trajectory groups. While gathering detailed information on the group with the highest caries experience have been challenging and difficult, this information is important for both professionals and patients, to better understand how to best prevent caries and stop its progression.

Big data like the SKaPa may be important tools for creating new codes for different treatment methods, providing opportunities to evaluate the effectiveness of preventive strategies over time [24]. This may be especially useful for high trajectory groups. This approach is also important when updating clinical practice guidelines, especially given the lack of randomised controlled trials and ethical problems with conducting such studies of caries disease [25].

Identifying individuals with the greatest caries experience may also better explain their treatment needs and facilitate development of more efficient preventive treatments, tailored to this patient group [26]. A thorough survey and investigation of high caries experience groups may also help to explain the underlying causes of caries disease and provide guidance for oral care planning and targeted preventive efforts.

Thus, the aim of this study was to describe the caries experience from a life course perspective, across seven age cohorts during a 10-year period using the three-trajectory caries model. Our hypothesis was: The same three-trajectory pattern [16, 17, 20] that identifies individuals with different caries experiences over time is reflected within each age cohort.

## Materials and methods

**Figure 1:**
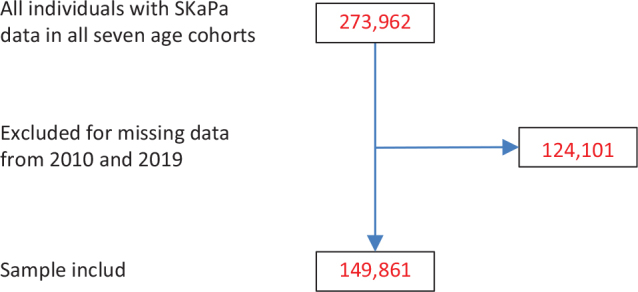
Flow chart of numbers of individuals of eligible (with SKaPa data), excluded (missing SKaPa data for 2010 and 2019), and included in analyses.

Longitudinal caries data were retrieved from the SKaPa, and then classified into seven age cohorts. The study included registry data from 2019 including 273,962 individuals aged 30–90 years whose records contained caries data (Figure 1). Among these, 124,101 individuals did not have a dental examination in both 2010 and 2019. The final 149,861 individuals included in analyses were recall patients at 1,333 general practice dental clinics in Sweden, and they covered most of Sweden’s 23 regions. Study data for each age cohort were retrieved retrospectively, for 10 years (i.e. 2010–2019). The numbers of individuals in each age cohort are shown in Table 1.

**Table 1:** Number of individuals in each age cohort (30–90 years) of SKaPa registry in each caries trajectory.

Age cohort, years*	30	40	50	60	70	80	90	Total
High (15%)	6,237	3,299	4,012	3,756	3,124	1,697	452	22,577
Moderate (45%)	20,081	10,619	12,814	12,074	9,238	4,988	1,327	71,141
Low (40%)	17,172	8,763	10,124	9,035	6,768	3,429	852	56,143
Total	43,490	22,681	26,950	24,865	19,130	10,114	2,631	149,861

* Age in 2019.

### Primary outcome variable

The main variable of interest was the DMFS index, which is based on a range of 0–32 teeth, and a maximum of 148 tooth surfaces.

### The model

The group-based trajectory modelling used in the Dunedin cohort [16], is a specialised application of finite mixture modelling. This approach simplifies analyses of longitudinal data by identifying developmental trajectory groups on a likelihood basis; this involves approaching a set of individual trajectories by grouping those that closely resemble one another using a probability function. Dealing with few trajectories is less complicated than analysing several hundred individual trajectories [16, 27]. In this study, a similar model was used just adapting the obtained proportions from the analysis of the Dunedin cohort [16]. A reason for this was to be able to compare and evaluate outcome from different populations in a better way.

The mean DMFS index values for each age cohort were assigned to three different trajectories during 2019: high (15% of sample), moderate (45% of sample), and low (40% of sample).

### Analysis

The mean DMFS index value for each trajectory was calculated at the start (2010) and end (2019) of the study period, and used to quantify the caries experience over time. The mean increase in DMFS across the study period was the main variable used to quantify caries activity for each trajectory. Two additional variables of interest were the mean numbers of decayed teeth (DT) and decayed tooth surfaces (DS) during the study period. Caries development patterns were compared between the trajectories within each age cohort as well as across all age cohorts for the entire dataset.

The Swedish Ethical Review Authority approved the research project (Dnr 2022-01689-02).

### Statistics

Descriptive statistics were used to describe caries development during the study period. Mean values were compared at the group level using the dependent-sample t- test. Because of the large sample sizes, we adopted a more conservative significance level thus *p*-values of less than or equal to .01 were considered significant.

To help in the interpretation of the results, we also calculated the effect size, which is a standardised measure of the strength of the relationship between, for example, two variables. When two variables are compared, it is appropriate to calculate Cohen’s *d* effect size [28], which is calculated by dividing the mean difference by the pooled standard deviation. To interpret the effect size *d*, the values suggested by Cohen were used: 0.2 = small effect, 0.5 = medium effect, and 0.8 = large effect.

Data were analysed using IBM SPSS Statistics version 28 for Windows (IBM Corporation; Armonk, NY, USA).

## Results

A total of 149,861 individuals were included in the study. The proportions within the three caries trajectories (15%, 45%, and 40%) are shown in Table 1. Mean DMFS values at the start (2010) and end (2019) of the study period for the trajectory groups in each of the seven cohorts (age range: 30–90 years) and the total sample are shown in Table 2.

**Table 2:** Mean DMFS index values at the start (2010) and end (2019) of the study period in the three caries trajectories for each age cohort (30–90 years) of SKaPa registry.

	High (15%)	Moderate (45%)	Low (40%)	Total

Age cohort, years*	Mean ± SD	Mean ± SD	Mean ± SD	Mean ± SD
**30**				
DMFS 2010	21.9 ± 9.7	8.2 ± 5.4	1.5 ± 2.9	7.5 ± 8.6
DMFS 2019	29.8 ± 11.0	10.5 ± 4.6	1.6 ± 1.4	9.8 ± 10.6
**40**				
DMFS 2010	32.3 ± 13.2	14.3 ± 6.2	3.6 ± 3.2	12.8 ± 11.7
DMFS 2019	43.5 ±16.1	17.7 ± 5.6	4.2 ± 2.9	16.1 ± 14.0
**50**				
DMFS 2010	45.5 ± 15.7	23.8 ± 7.6	9.0 ± 4.7	21.6 ± 15.0
DMFS 2019	57.4 ± 16.5	28.1 ± 6.7	10.5 ± 4.7	25.9 ± 17.6
**60**				
DMFS 2010	68.6 ± 19.5	40.4 ± 10.2	19.1 ± 7.8	36.9 ± 20.1
DMFS 2019	81.6 ± 17.4	46.4 ± 8.5	22.0 ± 7.5	42.9 ± 22.2
**70**				
DMFS 2010	96.6 ± 22.4	65.3 ± 14.3	36.8 ± 13.4	60.4 ± 25.8
DMFS 2019	111.3 ± 13.6	73.4 ± 10.2	41.5 ± 12.1	68.3 ± 26.4
**80**				
DMFS 2010	115.6 ± 23.1	86.1 ± 17.0	53.0 ± 17.8	79.9 ± 28.6
DMFS 2019	128.1 ± 8.5	94.2 ± 10.8	58.5 ± 15.1	87.8 ± 27.1
**90**				
DMFS 2010	122.8 ± 22.4	96.9 ± 16.7	62.5 ± 19.5	90.2 ± 28.4
DMFS 2019	134.0 ± 5.1	104.9 ± 10.5	68.0 ± 15.7	97.9 ± 26.0

DMFS: decayed, missing, and filled tooth surfaces; SD: standard deviation.

* Age in 2019.

The mean DMFS index increase during the 10-year study period was significant for all but one of the 21 trajectories across the age cohorts (Table 3). The DMFS index increase was significantly higher in the high trajectory group compared with the low and moderate trajectory groups within the same age cohort. Cohen’s *d* effect sizes were generally higher in the moderate and high trajectory groups compared with the low trajectory group, reflecting a larger increase in DMFS indexes in both of those groups. The effect sizes were medium to large according to Cohen’s guidelines for interpretation [28].

**Table 3:** Mean increase (*d* ± *sd*) in DMFS from the start (2010) to end (2019) of the study period in each caries trajectory for each age cohorts from the SKaPa registry.

	High (15%)		Moderate (45%)		Low (40%)		Total	

Age cohort, years*	*d* ± *sd*	*d*	*d* ± *sd*	*d*	*d* ± *sd*	*d*	*d* ± *sd*	*d*
**30**								
	7.9 ± 10.6***	0.75	2.4 ± 4.2***	0.56	0.01 ± 2.7 *n.s.*	0.01	2.2 ± 5.8***	0.39
**40**								
	9.4 ± 11.6***	0.81	3.5 ± 4.4***	0.79	0.8 ± 2.3***	0.34	3.3 ± 6.2***	0.53
**50**								
	10.9 ± 14.0***	0.78	4.3 ± 5.0***	0.85	1.5 ± 2.7***	0.55	4.2 ± 7.3***	0.58
**60**								
	13.0 ± 16.1***	0.81	6.0 ± 7.1***	0.85	2.9 ± 4.4***	0.67	5.9 ± 9.0***	0.66
**70**								
	14.6 ± 20.5***	0.71	8.1 ± 11.5***	0.70	4.7 ± 7.6***	0.62	7.9 ± 12.8***	0.62
**80**								
	12.5 ± 22.0***	0.57	8.1 ± 14.0***	0.58	5.4 ± 10.8***	0.50	7.9 ± 14.9***	0.53
**90**								
	11.2 ± 21.9***	0.51	7.9 ± 13.6***	0.58	5.5 ± 13.3***	0.42	7.7 ± 15.4***	0.50

*** *p* < 0.001.

* Age in 2019.

DMFS = decade, missing and filled tooth surfaces.

*d* ± *sd* = difference between registrations year 2010 and 2019 including the standard deviation.

*d* = Cohen’s effect size

**Figure 2:**
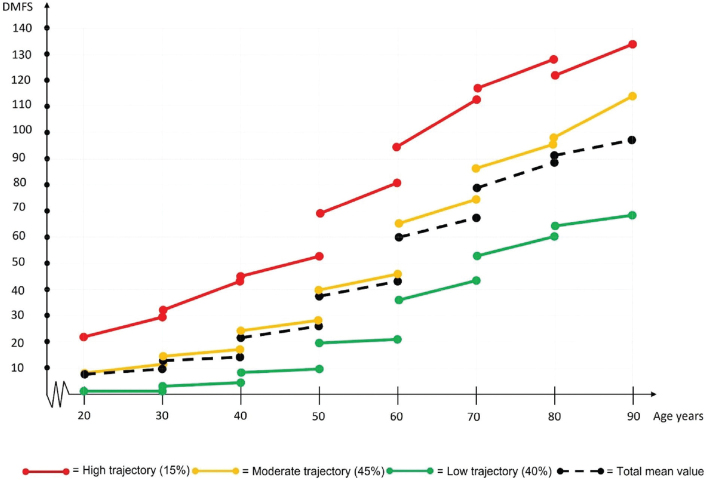
Progression of DMFS indexes for three different trajectories each age cohort during the study period (2010–2019). DMFS: decayed, missing and filled tooth surfaces.

The DMFS index increased steadily in the highest trajectory group for all age cohorts (Table 3 and Figure 2). A peak was observed for those in their 70s, after which it decreased for those in their 80s and 90s. In the moderate trajectory group, a larger DMFS index increase occurred for those in their 60s, 70s, and 80s. This increase was twice that of the moderate trajectory group in the younger age cohorts. A larger increase was also observed in the low trajectory group among those in their 70s, 80s, and 90s, although the increase was smaller compared with the other trajectories. Slightly different patterns of increase in the DMFS index were observed for the three trajectories: Cohen’s *d* effect sizes peaked for those in their 40s, 50s, and 60s in the high trajectory group, for those in their 50s and 60s in the moderate trajectory group, and for those in their 60s and 70s in the low trajectory group (Table 3).

Changes in caries experience over time between age cohorts were also compared, (i.e. one age cohort during 2010 was compared with a younger age cohort when they were at the same age during 2019). For all compared cohorts, a general decrease in caries experience was seen between 2010 and 2019, except for the high trajectory group in the oldest age group (those in their 90s) (Table 3 and Figure 2). The largest mean decrease in caries experience was for those in their 60s. The DMFS index was 60.4 ± 25.8 for the 70-year age cohort (age 60 years in 2010) and 42.9 ± 22.2 for the 60-year age cohort (age 60 years in 2019). Annual development of DS and DT within the three caries trajectories across each of the seven cohorts during the study period, are shown in Table 4.

**Table 4:** Mean decayed tooth surfaces (DS) and decayed teeth (DT) per year in each caries trajectory for each age cohort (30–90 years) from the SKaPa registry during the study period (2010–2019).

Age cohort, years*	High (15%)	Moderate (45%)	Low (40%)	Total
**30**				
DS/year	0.45 ± 0.10	0.15 ± 0.04	0.03 ± 0.01	0.21 ± 0.19
DT/year	0.38 ± 0.09	0.14 ± 0.04	0.03 ± 0.01	0.18 ± 0.16
**40**				
DS/year	0.39 ± 0.16	0.16 ± 0.06	0.05 ± 0.02	0.20 ± 0.17
DT/year	0.32 ± 0.12	0.14 ± 0.05	0.05 ± 0.02	0.17 ± 0.14
**50**				
DS/year	0.42 ± 0.08	0.17 ± 0.05	0.07 ± 0.02	0.22 ± 0.16
DT/year	0.33 ± 0.06	0.15 ± 0.04	0.06 ± 0.02	0.18 ± 0.12
**60**				
DS/year	0.34 ± 0.09	0.14 ± 0.04	0.07 ± 0.02	0.18 ± 0.13
DT/year	0.25 ± 0.06	0.12 ± 0.04	0.06 ± 0.02	0.14 ± 0.09
**70**				
DS/year	0.30 ± 0.10	0.16 ± 0.07	0.10 ± 0.05	0.19 ± 0.11
DT/year	0.22 ± 0.06	0.13 ± 0.05	0.08 ± 0.04	0.14 ± 0.08
**80**				
DS/year	0.27 ± 0.07	0.19 ± 0.09	0.13 ± 0.08	0.19 ± 0.10
DT/year	0.19 ± 0.04	0.15 ± 0.06	0.11 ± 0.07	0.15 ± 0.06
**90**				
DS/year	0.31 ± 0.05	0.26 ± 0.12	0.18 ± 0.13	0.25 ± 0.12
DT/year	0.20 ± 0.02	0.19 ± 0.08	0.15 ± 0.09	0.18 ± 0.07

Values are expressed as mean ± SD.

* Age in 2019.

## Discussion

Although dental caries was observed across the 10-year study in all age cohorts, it was significantly more severe in the high trajectory group within each age cohort. In these data from ~149,000 adults aged 30–90 years, the group with the highest caries experience also demonstrated an increased caries burden with age compared with the moderate and low trajectory groups.

A previous study using the three-trajectory caries model with SKaPa data [20] found similarities to the Dunedin study [16, 17]. The aim of this study herein was to replicate that method to analyse data from seven adult age cohorts with a larger, nationwide SKaPa sample. These analyses showed similar results, with the model able to identify groups with the highest caries experience over time in all age cohorts.

A steady DMFS index progression was observed with increasing age, especially in the highest trajectory groups. This DMFS index increase peaked among those in their 70s, after which it levelled off when individuals were in their 80s and 90s. Empirical evidence to guide treatment of dental caries in older adults is scarce compared with that for the treatment of children and adults with coronal caries. The finding herein suggests that improved caries prevention for the highest trajectory group is needed across all age cohorts.

The SKaPa data have been well validated, have satisfactory reliability and accuracy regarding dental caries among children aged 6–12 years, and are considered a reliable source for registry-based research [29, 30].

The SKaPa includes most of the Swedish population (7.4 million of 10 million inhabitants), across a wide range of general dental clinics, including both public and private, which are located throughout Sweden. The sample analysed herein should thus offer very good generalisability of these findings to the broader Swedish populations.

Further on, the three-trajectory caries model used in this study could be useful to identify groups with the highest caries experience and compare populations in SKaPa on national as well as regional and local levels.

This study was not without limitations. Use of the three-trajectory caries model with similar trajectory proportions, based on the Dunedin study, may have been influenced by variations in caries prevalence in different subgroups. Further analyses will be needed to identify alternative trajectories.

When comparing DMFS index values, reasons for the ‘missing’ component vary, and may be difficult to ascertain. They may also be essential to understanding this complex system [31]. Herein, we could not determine reasons for ‘missing’ component, though the SKaPa has previously described causes across ages [10]. Until age 40, caries is the most common reason for extractions. After age 40, pulp conditions and fracture, along with caries, account for >50% of extractions. After age 50, periodontitis accounts for 30% of the missing component. The number of annual extractions increases by age from around 5% in the youngest adult group to around 15% among those in their 90s [10].

Changes in caries experience over time should be considered when comparing longitudinal caries data from different time periods. Herein, a general decrease in caries experience was seen between 2010 and 2019 and was most pronounced among those in their 60s. This decrease may reflect a general increase in use of fluoride products. In 2008, the need for a prescription to buy 0.2% sodium fluoride (NaF) solutions was removed in Sweden, and then the 2011 National Guidelines recommended 0.2% NaF solution use for adults at risk for caries or with early caries that may progress [32]. The number of dispatches of prescribed fluorides from pharmacies was 544,964 in 2010, which increased to 618,226 in 2019 (including 0.2% NaF rinsing solutions and toothpaste with 5,000 ppm fluoride). Though information about over-the-counter fluoride products is more difficult to obtain, data from one of the largest manufacturers reported that 260,000 litres of 0.2% NaF rinsing solutions were sold in 2010, which increased to 2,213,000 litres in 2019. The consequences of this increase on caries development are difficult to determine; however, they might be described by the Common-Sense Model concerning patients’ self-management of healthcare and a system for creating action plans and implementing actions [33]. Individuals are more likely to continue a treatment they perceive to be effective, like arresting caries progression after many years of caries experience.

DMFS indexes differed significantly among the three trajectory groups at start of the study period, even within the youngest age group (Figure 2). This indicates that caries disease begins in younger age [34–36] and then continues, possibly for all cohorts. However, this pattern needs further investigation, by including even younger cohorts.

When longitudinal caries data are unavailable, cross-sectional studies of different age groups can provide information about caries development over the life course. Spending on fee-for-service dental claims and medical spending on oral health care for patients aged 0–89 years have been analysed using very large cross-sectional data sets from the USA-based Medicare and Medicaid programmes [37]. In both systems, average spending among adults increased to a peak at age 65 years, followed by a decrease. Our data showing an increase in caries experience among older groups are consistent with these increases in spending on oral treatments. Recent global demographic and epidemiological changes, such as the increased proportion of older individuals with more remaining teeth, will likely increase the need for targeted treatments for older adults. Although when and how to intervene more effectively with older groups have been discussed, further research is needed. Significantly increased management of root caries through use of highly fluoridated toothpastes or varnishes as well as antimicrobial agents, have been described [38]. Other barriers include better oral health cost coverage, general and oral health comorbidities, patient misconceptions and fear, along with needs for dental health system education [39].

The trend toward increased tooth retention and caries risk factors within the ageing population suggest a possible upsurge in the total burden of caries disease for this demographic group. This will likely pose a challenge for policymakers in designing oral health systems [40]. Studies have described increasing medication prescription for older; this is an issue because many drugs cause hyposalivation as a common side effect, which may increase caries risk [41–45].

Difficulties with reimbursement for preventive oral health measures across healthcare systems have been discussed [46], though there is insufficient evidence to determine whether current preventive interventions improve oral health in those with caries [25, 47]. This may explain why neither Medicare nor Medicaid incentivises adult preventive care [37], and why annual SKaPa data show that caries preventive measure use increases parallel with increased prevalence of fillings, except in the oldest age groups, despite government subsidisation [10]. Older adults may even choose extraction before paying for prevention [48]. This context may also explain why caries experience progressed with age herein, and suggests that effective preventive strategies have not reached those at highest caries risk in Sweden.

In conclusion, the findings herein support our hypothesis that the same three-trajectory pattern identifying those with the greatest caries experience over time is reflected in all age cohorts. The three-trajectory model used here to identify caries experience also appears to be useful for longitudinal studies. Caries experience increased over time within all cohorts, and particularly within the highest trajectory groups within the older cohorts. These trends demand that greater attention be paid to these groups and call for more efficient caries prevention methods.

## Data Availability

Study data are available on request to the authors.
